# Conventional Rapid Latex Agglutination in Estimation of von Willebrand Factor: Method Revisited and Potential Clinical Applications

**DOI:** 10.1155/2014/850810

**Published:** 2014-12-25

**Authors:** Marianor Mahat, Wan Zaidah Abdullah, Che Maraina Che Hussin

**Affiliations:** ^1^Departments of Haematology, School of Medical Sciences, Universiti Sains Malaysia, Health Campus, 16150 Kubang Kerian, Kelantan, Malaysia; ^2^Department of Immunology, School of Medical Sciences, Universiti Sains Malaysia, Health Campus, 16150 Kubang Kerian, Kelantan, Malaysia

## Abstract

Measurement of von Willebrand factor antigen (VWF : Ag) levels is usually performed in a specialised laboratory which limits its application in routine clinical practice. So far, no commercial rapid test kit is available for VWF : Ag estimation. This paper discusses the technical aspect of latex agglutination method which was established to suit the purpose of estimating von Willebrand factor (VWF) levels in the plasma sample. The latex agglutination test can be performed qualitatively and semiquantitatively. Reproducibility, stability, linearity, limit of detection, interference, and method comparison studies were conducted to evaluate the performance of this test. Semiquantitative latex agglutination test was strongly correlated with the reference immunoturbidimetric assay (Spearman's rho = 0.946, *P* < 0.001, *n* = 132). A substantial agreement (*κ* = 0.77) was found between qualitative latex agglutination test and the reference assay. Using the scoring system for the rapid latex test, no agglutination is with 0% VWF : Ag (control negative), 1+ reaction is equivalent to <20% VWF : Ag, and 4+ reaction indicates >150% VWF : Ag (when comparing with immunoturbidimetric assay). The findings from evaluation studies suggest that latex agglutination method is suitable to be used as a rapid test kit for the estimation of VWF : Ag levels in various clinical conditions associated with high levels and low levels of VWF : Ag.

## 1. Introduction

von Willebrand factor (VWF), a multimeric glycoprotein produced by endothelial cells and megakaryocytes, plays important roles in platelet adhesion at the sites of vascular injury and in the coagulation process through stabilization of factor VIII [1–3]. VWF was first identified in hereditary bleeding disorder known as von Willebrand disease (VWD) and later it has been associated with other clinical conditions such as cancers, clotting, and vascular and liver disorders. Quantitative and functional impairment of VWF may lead to bleeding disorder. In contrast, elevated levels of VWF predispose to thrombotic complications [2, 3]. Epidemiological studies have revealed that an increased level of VWF is significantly associated with cardiovascular diseases and acute vascular events [4–8], making it a potential biomarker in disease progression and prognosis [9–11]. Cardiovascular disease is a global health problem and is a leading cause of death in developed countries [12]. In developing countries, cardiovascular disease has become increasingly prevalent [12, 13].

Currently, the available test for measurement of VWF level in plasma is VWF antigen (VWF : Ag) assay that is usually performed by enzyme-linked immunosorbent assay (ELISA) and automated immunoturbidimetric procedure such as latex-immunoassay (LIA), which requires sophisticated instrumentation and well-trained laboratory personnel. Furthermore, ELISA test is unsuitable for urgent testing [14, 15]. Despite the advanced technology of testing VWF levels in a fully equipped laboratory, very little attention has been focused on testing the VWF levels in areas with limited laboratory facilities or at the bedside. Thus, the application of rapid, simple, and less expensive test method for the detection of VWF might help in improving clinical management in resource-limited setting. In the future, estimation of VWF : Ag could be possible as a point of care testing in clinical practice routinely.

Latex agglutination test has been the method of choice in the development of a rapid test kit in many fields including clinical and veterinary medicines [16–20]. In principle, latex agglutination test is based on agglutination reactions between antigen and antibody. Submicron microspheres or often called “latex beads” are used as a solid support for the antibody (or antigen) to be adsorbed onto them. The latex beads with adsorbed antibody (or antigen) are used to detect antigen (or antibody) present in biological samples. Polystyrene latex beads are commonly used in the latex agglutination test because of their strong hydrophobic characteristic that is ideal for the adsorption of materials such as proteins by a simple passive adsorption method [21].

The use of latex beads was first described for the detection of rheumatoid factor by Plotz and Singer in 1956 [22]. Since then, latex tests have been developed to detect specific infectious diseases, autoimmune diseases, hormones, drugs, and serum proteins. The original method for attachment of proteins (antibody) to latex beads was passive adsorption. Passive adsorption using plain polystyrene latex beads has been used in the development of many latex tests for the detection of antibody or antigen such as Group D salmonellae [23], CRP [24], lactoferrin [25], and rotavirus [26]. Covalent coupling method using functionalized latex beads such as carboxylated polystyrene latex beads can produce more stabilized protein-latex complex compared to passive adsorption method. It has then become a method of choice in the development of latex tests such as for the detection of anti-cysticercus antibodies [27], IgM quantification in cerebrospinal fluid [28], and detection of avian influenza virus subtype H5N1 [29]. Although covalent coupling methods have many advantages, the passive adsorption method is still widely used until today because of its simplicity and flexibility.

Conventional latex agglutination is not a new method, but its application is limited in the field of hematology and hemostasis. The latex method has moved towards automation and currently the antigenic detection has been made easy and specific with this new technology especially using the monoclonal generated antibodies. However, the conventional method may be useful to be applied as a rapid or screening test before running the usual laboratory investigations. The present study may provide evidence on the usage of a latex agglutination test for the detection of VWF : Ag in the plasma sample that could be commercialized in the future. In this study, surfactant-free latex beads were selected in order to avoid a variation in protein attachment condition that might occur due to the variation in the surfactant purity. The aggregation in the latex beads is prevented by the electrical charge that is built onto the surface of the latex beads during their synthesis. Chloromethyl latex beads have been selected to be used in the passive adsorption method. The beads have a high density of chloromethyl groups attached to the styrene monomeric unit, and these functional groups can react directly with amino groups in antibodies. The beads are of hydrophobic type and can be used at both high and low pH conditions. The beads are stabilized by negatively charged sulfate groups that provide the colloid stability. In terms of ease of washing and suitability in simple visible test, latex beads with a size of 1 *μ*m were used.

This study was conducted with the aims to develop and validate a latex agglutination test for the detection of VWF : Ag. Method validation is a process to demonstrate the suitability of the test method for its intended purpose. Method validation is performed to determine the performance characteristics of the test method, for example, reproducibility, stability, linearity, limit of detection, and comparison of method, and also to estimate certain types of analytical errors by performing interference study. Reproducibility study estimates imprecision, stability study determines stability of the sample and reagent, linearity study determines reportable range, detection limit study determines the lowest concentration of analyte, method comparison estimates inaccuracy, and interference study estimates error caused by other materials that may be present in the specimen.

## 2. Materials and Methods

### 2.1. Sample Collection and Plasma Preparation

The study protocol was approved by the Human Research Ethics Committee from Universiti Sains Malaysia, Health Campus, Kubang Kerian, Kelantan, Malaysia [reference number: USMKK/PPP/JEPeM 253.3.(4)]. Blood samples for validation studies were collected into 3.2% buffered trisodium citrate (Becton Dickinson and Co., Plymouth, UK), and platelet-poor plasma was prepared by centrifuging the blood at 2,500 g for 15 minutes at room temperature. Following centrifugation, the top 2/3 of the plasma layer was transferred into a plastic tube. The collected plasma was recentrifuged at 2,500 g for 15 minutes to remove any remaining red cells or platelets. After centrifugation, the top 2/3 portion of plasma volume was transferred into cryogenic vials, and the plasma samples were frozen at −70°C until use. On the day of testing, plasma samples were thawed at 37°C for 10 minutes and mixed by gentle inversion prior to analysis, which was performed within two hours after thawing.

### 2.2. Preparation of VWF Antibody-Latex Reagent

Chloromethyl latex beads, 1.0 *μ*m in diameter, were purchased from Life Technologies, USA. Prior to use, the latex beads were washed according to the manufacturer's instructions with some modifications. Briefly, 2.5 mL of 4% latex suspension was washed twice in 10 mL imidazole buffer, pH 7.4 (120 mM NaCl, 20 mM imidazole, and 5 mM citric acid) by centrifuging the latex suspension at 3,000 g for 20 minutes at room temperature. The final pellet was resuspended with 5 mL imidazole buffer to obtain a suspension of 2%.

VWF antibody-latex reagent was prepared by passive adsorption method according to the manufacturer's protocol and the method described by Mina et al., 2012 [30], with some modifications. Briefly, 1.0 mL of 2% latex suspension was centrifuged at 3,000 g for 20 minutes at room temperature, and the supernatant was discarded. The pellet was resuspended with 1 mL phosphate-buffered saline, pH 7.4 (135 mM NaCl, 2.6 mM KCl, 8 mM Na_2_HPO_4_, and 1.5 mM KH_2_PO_4_), that was added with 200 *μ*L of polyclonal rabbit anti-human von Willebrand Factor from Dako Denmark (3.1 g/L). The mixture was incubated with gentle mixing on a horizontal rotator (Barnstead Thermolyne Labquake Rotator, USA) at room temperature for 24 hours. After incubation, the mixture was centrifuged, and the supernatant was kept for determination of unadsorbed antibody by BCA method using Micro BCA Protein Determination Kit from Pierce Biotechnology, USA. The concentration of unadsorbed antibody in the supernatant was determined according to the manufacturer's instructions. The concentration of adsorbed antibody was estimated as the difference between the initial concentration of antibody (i.e., 620 *μ*g) and the concentration of unadsorbed antibody in the supernatant.

The pellet was then washed twice in 4 mL imidazole buffer by centrifugation as stated earlier and resuspended with 3 mL imidazole buffer containing 1% bovine serum albumin (Sigma-Aldrich, USA). The mixture was incubated with gentle mixing at room temperature for one hour. After incubation, the mixture was centrifuged, and the pellet was washed twice in 4 mL imidazole buffer by centrifugation as stated earlier. The final pellet was resuspended with 2 mL imidazole buffer containing 0.1% bovine serum albumin to obtain a suspension of 1%, and the suspension was stored at 4°C until use.

The process described was carried out using different concentrations of the VWF antibody to determine the appropriate concentration of antibody for the adsorption procedure. As a control reagent, the process was conducted without the antibody. All reagents were tested on known negative and positive samples. Normal saline and buffers were used as negative samples while commercial controls were used as positive samples.

### 2.3. Latex Agglutination Test Procedure and Result Interpretation

VWF antibody-latex reagent was brought to room temperature and mixed gently before use. The sedimentation of latex beads can occur during storage that can be reversed by pipetting the suspension of latex beads through a fine tip pipette until they are returned to a uniform suspension. Latex agglutination test for the detection of VWF in a plasma sample was performed on a slide card (Thermo Scientific, UK). Using a wooden applicator stick, 20 *μ*L of VWF antibody-latex reagent was mixed with 20 *μ*L of plasma sample on a black reaction zone of the card. The card was rotated on a horizontal shaker (Boekel Grant Microplate Shaker, UK) for three minutes. After three minutes, the agglutination reaction was observed by visual inspection macroscopically with a naked eye.

**Figure 1:**
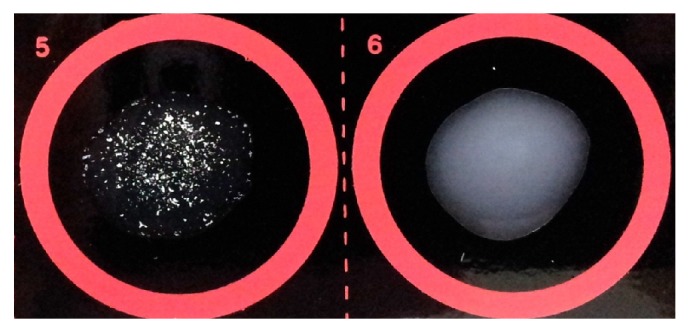
Positive (number 5) and negative (number 6) reactions of qualitative latex agglutination test for VWF : Ag.

The detection and estimation of VWF by latex agglutination test can be performed using a qualitative test and semiquantitative test, respectively. Qualitative test was performed on undiluted plasma sample, and the result was reported as negative or positive. Invisible or hardly visible agglutination was reported as negative and visible agglutination was reported as positive (Figure 1). For a positive result, the degree of agglutination was scored from 1+ to 4+ based on the size of the agglutinated beads, the appearance of the background, and the rapidity of the agglutination development as shown in Table 1. Semiquantitative test was performed on diluted plasma sample. A positive plasma sample was serially diluted twofold with normal saline. The highest dilution that still shows a positive reaction is the end-titre of a sample.

### 2.4. Validation Studies on VWF Antibody-Latex Reagent

#### 2.4.1. Reproducibility Study

Reproducibility studies were performed within a day and between days. Within-day reproducibility study was carried out by testing VWF antibody-latex reagent on three plasma samples, and the end-titre of each sample was determined three times. Between-day reproducibility study was conducted by testing the reagent in duplicate on two plasma samples, and the end-titre of the samples was determined on days 1, 7, 14, and 21. The reagents were also tested on low and normal controls from Instrumentation Laboratories, Italy (Hemosil Special Test Control Level 2 and Hemosil Normal Control Assayed, resp.), on days 2, 4, 5, 7, 30, and 50.

#### 2.4.2. Stability Study

Stability of the reagent was checked during storage of VWF antibody-latex reagent at 4°C for 21 days. Qualitative test was performed on two plasma samples on days 1, 2, 3, 4, 5, 6, 7, 14, and 21 using aliquots of samples that were stored at −70°C. A new aliquot of sample was used in each experiment. Stability of plasma sample during storage was also checked by testing VWF antibody-latex reagent (stored at 4°C) on aliquots of plasma samples which was stored at 4°C. After 0 (within two hours after blood collection), 1, 2, 3, 4, 5, 6, 7, 14, and 21 days, the qualitative test was performed. The reagents were also tested on Hemosil Special Test Control Level 2 and Hemosil Normal Control Assayed (Instrumentation Laboratories, Italy). Semiquantitative latex agglutination test was performed on these controls for up to 50 days. Batch to batch variation was tested with the reagents prepared on two different dates, using two different lots of the VWF antibody.

#### 2.4.3. Linearity Study

The linearity of latex agglutination test was determined by testing VWF antibody-latex reagent on two patient samples which were serially diluted twofold. The VWF : Ag level in diluted samples was measured by immunoturbidimetric assay using Hemosil von Willebrand Factor Antigen kit (Instrumentation Laboratory, Italy). Semiquantitative latex agglutination test was performed on diluted samples, and the end-titres were compared with the VWF : Ag levels. Graphical presentations of the semiquantitative VWF : Ag by latex agglutination test (*Y*) versus the VWF : Ag by immunoturbidimetric assay (*X*) were made for individual sample and also for a combination of the two samples.

#### 2.4.4. Determination of Limit of Detection

Limit of detection of latex agglutination test was determined by testing VWF antibody-latex reagent on low-level samples (cryosupernatant). Seven cryosupernatant samples were serially diluted twofold, and the VWF : Ag levels in the samples (neat) were measured by immunoturbidimetric assay using Hemosil von Willebrand Factor Antigen kit (Instrumentation Laboratory, Italy). VWF : Ag levels in 1 : 2, 1 : 4, and 1 : 8 diluted samples were estimated by dividing the VWF : Ag levels of neat samples by the dilution factors of 2, 4, and 8, respectively. Latex agglutination test was performed to determine the lowest level of VWF : Ag that shows positive agglutination reaction.

#### 2.4.5. Interference Study

*(i) Interference from Hemoglobin*. Interference from hemoglobin is common in coagulation testing, and hemolytic specimen is usually not or minimally affected in latex agglutination test. The interference study was performed by testing VWF antibody-latex reagent on plasma pools and plasma pools spiked at different concentrations of hemolysate. Hemolysate was prepared by freezing and thawing the whole blood followed by the osmotic shock protocol [31]. Briefly, normal sample that was collected in 3.0 mL K_2_ EDTA tube was centrifuged at 1,000 g for 10 minutes. Plasma was removed and replaced with an equal volume of isotonic saline. Cells were resuspended, and the suspension was centrifuged again at 1,000 g for 10 minutes. The saline wash was repeated three times. In the final wash, saline was replaced with distilled water, and the suspension was stored at −20°C overnight. Frozen cells were thawed, mixed, and centrifuged at 2,000 g for 30 minutes to remove cell debris.

Hemolysate was transferred to a clean tube and hemoglobin level was measured with the Sysmex XE 5000 Hematology analyzer (Sysmex Corporation, Kobe, Japan). Stock solutions of hemolysate in normal saline were prepared, and each of the stock solutions was used to spike plasma pools with high and low VWF : Ag levels. In the present study, hemolysate with hemoglobin level of 120 g/L was used to prepare stock solutions of hemolysate. The hemolysate was diluted 1 : 2, 1 : 4, 1 : 8, 1 : 20, and 1 : 40 with normal saline to obtain the stock solutions with hemoglobin levels of 60, 30, 15, 6, and 3 g/L, respectively. One hundred microlitres of each stock solution were added to 900 *μ*L of plasma pools to obtain plasma pools with a final hemoglobin level of 6, 3, 1.5, 0.6, and 0.3 mg/mL.

*(ii) Interference from Rheumatoid Factor*. Rheumatoid factor (RF) is one of the most common interferences in latex-based methods. It may bind and cross-link antibodies attached to the latex beads, leading to a false-positive result or falsely elevated levels of VWF in a sample [32]. The effect of RF on agglutination reaction was studied by testing VWF antibody-latex reagent on plasma pools with high and low VWF : Ag levels. The plasma pools were spiked at different concentrations of RF using rheumatoid factor control serum from MyBioSource, USA (1030 IU/mL).

The RF control serum was diluted 1 : 8, 1 : 7, and 1 : 6 with high plasma pools to obtain plasma pools spiked with RF at a concentration of 129, 147, and 172 IU/mL, respectively. For the preparation of low plasma pools spiked with RF at a concentration of 10, 15, 52, 103, and 129 IU/mL, the RF control serum was diluted 1 : 100, 1 : 70, 1 : 20, 1 : 10, and 1 : 8, respectively, with low plasma pools.

#### 2.4.6. Method Comparison with the Reference Method

Method comparison study between latex agglutination test and the reference method (immunoturbidimetric assay) was conducted by parallel analysis of 132 plasma samples of different levels of VWF : Ag. The samples were obtained from 40 healthy individuals, 40 patients with cardiovascular diseases, and 32 patients with other diseases including malignant and inflammatory disorders. The remaining 20 samples were cryosupernatant samples (i.e., samples with low levels of VWF : Ag) that were obtained from Blood Transfusion Services. All specimens were tested in ten days, and the testing was spread over ten months.

Qualitative and semiquantitative latex agglutination tests were performed using the VWF antibody-latex reagents. The immunoturbidimetric assay was performed on ACL Elite Pro Coagulation analyser (Instrumentation Laboratory, Italy) using Hemosil von Willebrand Factor Antigen kit from Instrumentation Laboratory, Italy. The procedure for VWF : Ag by immunoturbidimetric assay was performed according to the manufacturer's instructions and adhered to the CLSI Document H21-A5 on the specimen collection and processing of blood specimens for testing the plasma-based coagulation assays. Normal reference range for VWF : Ag levels by immunoturbidimetric assay in our laboratory is 50–150%.

### 2.5. Statistical Analysis

Statistical analyses were conducted using the SPSS software version 20 (SPSS Inc., Chicago, IL, USA). Linear regression analysis was performed to determine the linearity of the semiquantitative latex agglutination test. The degree of agreement between qualitative latex agglutination test and immunoturbidimetric assay was estimated by calculating the kappa value (*κ*): 0.81–0.99, almost perfect agreement; 0.61–0.80, substantial agreement; 0.41–0.60, moderate agreement; 0.21–0.40, fair agreement; 0.01–0.20, slight agreement; and <0, less than chance agreement [33]. Spearman's rho correlation analysis was used to determine the degree of relationship between semiquantitative latex agglutination test and immunoturbidimetric assay. The strength of the relationship is showed by the correlation coefficient value (*r*): at least 0.8, very strong; 0.6 up to 0.8, moderately strong; 0.3 to 0.5, fair; and less than 0.3, poor [34]. Two-tailed probability (*P*) value of less than 0.05 was considered statistically significant.

## 3. Results

### 3.1. Preparation of VWF Antibody-Latex Reagent

Table 2 shows the estimated concentration of adsorbed antibody on the latex beads in the VWF antibody-latex reagents that were prepared using different concentrations of the VWF antibody. The results showed that the concentration of adsorbed antibody increased as the initial concentration of the VWF antibody increased. However, the percentage of adsorbed antibody, that is, 70% and 74%, was not much different when adsorption method was performed using antibody concentration of 620 *μ*g and 700 *μ*g, respectively.

Visual observation on the agglutination reaction showed that less agglutination was observed from latex beads adsorbed with less than 400 *μ*g of antibody (i.e., 118 *μ*g, 276 *μ*g, and 328 *μ*g) compared to agglutination reaction from latex beads adsorbed with about 400 *μ*g of antibody. Agglutination reactions from latex beads coated with 520 *μ*g antibody were slightly more compared to agglutination reactions from latex beads coated with 436 *μ*g antibody (data not shown). In other words, latex beads with ~400 *μ*g adsorbed antibody were sufficient to produce strong agglutination reaction when mixed with plasma sample. Thus, 200 *μ*L of the VWF antibody with a concentration of 620 *μ*g has been chosen to be the most appropriate concentration for the adsorption procedure.

Autoagglutination was not seen when VWF antibody-latex reagent alone was rotated for three minutes. Extending the time beyond three minutes resulted in reagent drying that might give false-positive result. No agglutination was seen when the control reagent (i.e., reagent without adsorbed antibody) was tested on plasma sample and commercial controls. Negative reaction was consistently observed when the VWF antibody-latex reagent was tested on normal saline and buffers throughout this study.

### 3.2. Validation Studies

Validation studies were conducted using VWF antibody-latex reagents that were prepared in seven batches. The concentration of adsorbed antibody on the latex beads ranged from 403 to 447 g (i.e., 65–72% from the initial concentration of the VWF antibody used in the adsorption procedure) which showed that the percentage of the VWF antibody adsorbed on the latex beads was consistent throughout this study.

#### 3.2.1. Reproducibility and Stability Studies

In the reproducibility study, the end-titre of three samples (i.e., 1 : 32, 1 : 64, and 1 : 128) was consistent when tested three times within a day. Similarly, the end-titre of two samples (i.e., 1 : 16 and 1 : 128) did not change from day 1 to day 21 (between days). Testing on the controls showed that the end-titre of low and normal controls (1 : 4 and 1 : 16, resp.) was reproducible from day 1 to day 50. In the stability study, VWF antibody-latex reagent and plasma sample were found to be stable for qualitative latex agglutination test when stored at 4°C for at least 21 days. Semiquantitative test on controls showed that the VWF antibody-latex reagent was stable for at least 50 days.

All these findings showed that the semiquantitative latex agglutination test results were reproducible within a day and between days and the reagent was found to be stable for at least 50 days when stored at 4°C. Batch to batch variation was not detected.

#### 3.2.2. Linearity Study

In the linearity study, a series of known concentrations of VWF : Ag was established by dilution with five equally spaced concentrations as shown in Table 3. The table shows the latex agglutination test results (titre) and the levels of VWF : Ag in undiluted (tube 1) and diluted samples (tubes 2–6). It was shown in the table that the semiquantitative latex agglutination test results (titre) were directly proportional to the levels of VWF : Ag in the plasma sample. Linearity of the semiquantitative VWF : Ag latex agglutination test was demonstrated in sample 1 (*r* ^2^ = 0.9985) and sample 2 (*r* ^2^ = 0.9999). A regression line that was fit to the points was observed in both samples (graphs not shown). The linearity was also demonstrated at the titre ranging from 1 : 2 to 1 : 128 when the results of both samples were combined and analyzed (*r* ^2^ = 0.9361). All these findings have demonstrated a linear relationship between the observed results (VWF : Ag titre) and the true concentrations of VWF : Ag in the plasma samples.

#### 3.2.3. Determination of Limit of Detection

In the limit of detection study, plasma samples with low levels of VWF : Ag (in the range of 20.2% to 30.6%) were obtained from seven cryosupernatant samples (Table 4). Table 4 shows the agglutination reaction observed when latex agglutination test was performed on undiluted (neat) samples and diluted samples (at 1 : 2, 1 : 4, and 1 : 8 dilutions). As shown in the table, the lowest level of VWF : Ag that still showed positive reaction is 5.1% (sample number 2 at 1 : 4 dilution), and the highest level of VWF : Ag that showed negative reaction is 3.8% (sample number 7 at 1 : 8 dilution). These results showed that the lowest level of VWF : Ag that the latex agglutination test can detect to determine the presence (positive result) or absence (negative result) of VWF : Ag was 5.1%. Thus, the limit of detection of VWF : Ag by latex agglutination test is approximately 5%. Besides that, reproducibility of the agglutination reaction at VWF : Ag levels of 5.1 and 3.8% was also checked by testing six times the diluted samples of number 2 (at 1 : 4 dilution) and number 7 (at 1 : 8 dilution), respectively. Sample number 2 showed positive reactions in all six replicates, while sample number 7 showed negative reaction in all six replicates. These findings showed that the latex agglutination test reliably produced consistent results near the cutoff concentration.

#### 3.2.4. Interference Study

Visible agglutination was seen when latex agglutination test was performed on undiluted plasma pools and undiluted plasma pools with a final hemoglobin level of 0.3, 0.6, 1.5, 3, 6, and 12 mg/mL. The end-titre of 1 : 32 was obtained when semiquantitative latex agglutination test was performed on high plasma pool. The same end-titre (1 : 32) was observed when the test was performed on high plasma pools with hemoglobin level of 0.3, 0.6, 1.5, 3, 6, and 12 mg/mL. Similarly, the end-titre of low plasma pools with hemoglobin level of 0.3, 0.6, 1.5, 3, 6, and 12 mg/mL was the same as the end-titre of low plasma pool without hemoglobin interference (1 : 4). These results showed that hemoglobin did not cause interference in latex agglutination test and semiquantitative test result was not affected by hemoglobin level up to 12 mg/mL.

Table 5 shows the end-titre of high and low plasma pools spiked at different concentrations of RF. As shown in the table, interference from RF on latex agglutination test was not detected in high plasma pools spiked with RF at concentrations up to 129 IU/mL. Nevertheless, the interference from RF was observed in low plasma pools spiked with RF at concentrations of 26, 52, 103, and 129 IU/mL. The end-titre of high and low plasma pools without interference from RF was 1 : 64 and 1 : 2, respectively. These results showed that RF interfered with the latex agglutination test result when the test was performed on the plasma sample containing low levels of VWF : Ag and high levels of RF (>26 IU/mL). However, latex agglutination test result of plasma sample containing high levels of VWF : Ag was not affected by RF at concentrations of <130 IU/mL.

#### 3.2.5. Method Comparison with the Reference Method

Comparison study between qualitative latex agglutination test and immunoturbidimetric assay showed a kappa value of 0.77 which indicates a substantial agreement between the two tests.  Table 6 shows the crosstabulation of 132 samples included in this study. Semiquantitative latex agglutination test was strongly correlated with immunoturbidimetric assay (Spearman's rho, *r* = 0.946, *P* < 0.001) across a range of VWF : Ag levels from 34.5% to 870%.

Based on the results from kappa statistic and correlation study, the scoring system for agglutination reactions and correlation with the VWF : Ag levels (using immunoturbidimetric assay) is shown in Table 7. The proposed interpretation guide is also put in a footnote.

## 4. Discussion

To the best of our knowledge, there has been no reported study or technical description available on the rapid test for VWF : Ag utilising latex agglutination method. At present, the application of this method has not been used in clinical practice for monitoring of patients with low and high plasma levels of VWF : Ag. Screening of VWD patients before confirmation tests and monitoring of patients at high risk of thrombotic event in the area with low-resource settings or at the bedside can be performed if rapid test for VWF : Ag is available. Hence, there is a need to develop a rapid and simple latex agglutination test that can reliably detect and estimate VWF : Ag in the plasma sample.

Latex agglutination test is based on the observation of visible clumps that are formed from the reaction of antigen-antibody complexes. The antigen-antibody complexes can be prepared by passive adsorption or covalent coupling method. In the early stage of the present study, attempts had been made to couple VWF antibody with the carboxylated latex beads by covalent coupling method. The coupling procedure was performed in MES buffer at pH 6.0 according to the protocol from the manufacturer. Unfortunately, there was no antibody coated on the latex beads (data not shown). However, the VWF antibody was successfully adsorbed onto the surface of chloromethyl latex beads by passive adsorption method. These findings were similar to the reported study by Garcia et al., 2015 [35]. In the present study, a maximal physical adsorption of the VWF antibody onto chloromethyl latex beads was obtained at pH 7.4, a pH that is close to the isoelectric point of polyclonal immunoglobulin (IgG) antibody [36, 37].

Interference by RF in the latex agglutination test for the detection of VWF : Ag was expected. RF is known to cause analytical error in automated immunoturbidimetry assay that presented the main limitation of this method. Despite this limitation, the findings do nevertheless show that the effect of the interference was less prominent in the sample with high levels of VWF : Ag compared to the sample with low levels of VWF : Ag. The expected interference is without any doubt due to the usage of whole IgG in the passive adsorption method. It is well known that rheumatoid factor can bind with the Fc region of IgG, causing false agglutination that may alter the result of the test. In the literature, the usage of antibody fragments such as Fab or F(ab′)_2_ was recommended to eliminate the interference from rheumatoid factor [37]. Antibody fragment is suggested in future study, and optimization is required as the antibody fragment is more acidic than the polyclonal antibody.

In the present study, the agglutination observed from the reaction between VWF antibody-latex reagent and positive sample may not be optimum as expected. Experiments using higher initial concentration of VWF antibody with a more intensive procedure to ensure maximal bead coating could be performed in the future study. Not only that, but the orientation of adsorbed antibody could also be studied to ensure a proper alignment of antibody on latex beads. Clarizia et al., 2009 [38], have demonstrated a method for detecting the aligned and misaligned antibodies on the surface on latex beads. Due to some technical constraint, maximal bead coating cannot be ensured, and a study on orientation of adsorbed antibody could not be performed. Another limitation of this study is that the size of the clumping was only estimated by visual observation. In future study, it is recommended to measure the clumping or macroscopic clusters using microscope or other methods.

There is a potential clinical application of this rapid latex agglutination test as a routine test in various premises including small laboratory and clinic and at the bedside. The clinical aspect of low levels of VWF : Ag is well recognized, but not many medical personnel realized the implications of high VWF : Ag levels in medical practice. An inherited bleeding disorder known as von Willebrand disease (VWD) is associated with an abnormality in VWF, and type 1 VWD is the commonest form that is associated with low VWF : Ag [39]. On the other hand, high levels of VWF : Ag are associated with thrombotic risks and had been extensively studied in many medical conditions particularly coronary heart disease (CHD) and stroke. It has been shown that CHD patients with high baseline of VWF : Ag levels are at risk of acute vascular events related to thrombosis [40]. The current clinical guidelines do not incorporate the measurement of VWF : Ag in the routine assessment of CHD patients but, from various studies, there is a role in monitoring the levels of VWF : Ag. Detection of persistent high levels or increasing trends of VWF : Ag levels would alert the treating doctor for certain clinical measures to improve the patient care.

Latex agglutination test for the detection of VWF : Ag was found to be simple to perform, and the results can be obtained within three minutes, making it suitable to be used in areas with limited laboratory facilities. The result obtained by the latex agglutination test is reliable as shown by the findings from reproducibility, stability, linearity, limit of detection, and method comparison studies. Prozone effect was not detected at VWF : Ag levels up to 870%. The stability of the reagents used for this method was up to 50 days and in this study no stabilizer or preservatives were used. The plasma for VWF : Ag estimation was stable for up to 21 days which may be related to the protein structure; however, more tests are needed to confirm the suitable plasma storage recommendation for this method. All these findings showed the usefulness of this method in detecting and estimating high and low levels of VWF : Ag. This method might be useful in the monitoring of VWF : Ag in patients that are at a high risk of vascular event related to endothelial dysfunction. Similarly, this method could be used to estimate VWF : Ag levels in patients with VWD before confirmation with standard test panels in the specialized coagulation laboratory.

However, we only evaluate the latex agglutination test in quantitative terms by using plasma samples containing low and high levels of VWF : Ag. This application mainly addressed the VWD type 1 which is more common in clinical practice. In future study, plasma samples with qualitative VWF abnormalities (e.g., type II VWD or acquired VWD patients demonstrating abnormal VWF function with variables VWF : Ag levels) could be included in the comparison study to confirm the application of the latex agglutination test for assessing VWF : Ag levels in various clinical situations. In summary, this test method needs to be refined and compared with other latex agglutination tests available in the market before it is used on patient samples.

## 5. Conclusion

Latex agglutination test for VWF : Ag is simple, rapid, and reproducible, correlates well with the reference method, and is suitable to be used in areas with limited laboratory facilities. The test has commercial potential as a low-cost alternative method for the detection and estimation of low levels and high levels of VWF : Ag that could help in the management of VWD type 1 patients and patients at risk of thrombotic events, respectively. However, the test may not be suitable for patients with a high level of rheumatoid factor.

## Figures and Tables

**Table 1:** Test score for qualitative latex agglutination test for VWF : Ag.

Test score	Size of the agglutinated beads	Appearance of the background	Rapidity of the agglutination
1+	Small clumps	Cloudy	>2 min
2+	Small clumps	Cloudy	>1 min
3+	Small/large clumps	Clear	>30 sec
4+	Large clumps	Clear	<30 sec

VWF : Ag: von Willebrand factor antigen.

**Table 2:** Estimated concentration of adsorbed antibody in the VWF antibody-latex reagents.

Initial volume of antibody (*μ*L)	Initial concentration of antibody (*μ*g)	Concentration of unadsorbed antibody (*μ*g)	Concentration of adsorbed antibody (*μ*g)	Percentage of adsorbed antibody (%)
100	310	192	118	38
150	465	189	276	59
162	502	174	328	65
200	620	184	436	70
226	700	180	520	74

VWF: von Willebrand factor.

**Table 3:** Linearity study on semiquantitative VWF antigen by latex agglutination test.

Tube number	Sample 1		Sample 2	
	VWF : Ag (%)^a^	VWF : Ag (titre)^b^	VWF : Ag (%)^a^	VWF : Ag (titre)^b^
1	600	1 : 128	460	1 : 64
2	300	1 : 64	230	1 : 32
3	150	1 : 32	115	1 : 16
4	86.9	1 : 16	57.9	1 : 8
5	22.2	1 : 8	27.9	1 : 4
6	9.9	1 : 4	17.6	1 : 2

^a^VWF : Ag levels (%) measured by immunoturbidimetric assay using Hemosil von Willebrand Factor Antigen kit from Instrumentation Laboratory, Italy.

^b^VWF : Ag (titre) estimated by using semiquantitative latex agglutination test.

VWF: von Willebrand factor; VWF : Ag: von Willebrand factor antigen.

**Table 4:** Limit of detection of VWF antigen detected by using latex agglutination test.

Sample/sample number	Neat		1 : 2		1 : 4		1 : 8	
	VWF : Ag (%)^a^	LAT^e^	VWF : Ag (%)^b^	LAT^e^	VWF : Ag (%)^c^	LAT^e^	VWF : Ag (%)^d^	LAT^e^
1	24.2	+	12.1	+	6.1	+	3.0	−
2	20.2	+	10.2	+	5.1	+	2.5	−
3	30.6	+	15.3	+	6.9	+	3.8	−
4	27.6	+	13.8	+	6.9	+	3.4	−
5	26.9	+	13.3	+	6.7	+	3.4	−
6	29.1	+	14.6	+	7.3	+	3.6	−
7	30.3	+	15.2	+	7.6	+	3.8	−

^a^VWF : Ag levels in neat sample that was measured by immunoturbidimetric assay using Hemosil von Willebrand Factor Antigen kit from Instrumentation Laboratory, Italy.

^b,c,d^Estimated VWF : Ag levels in 1 : 2, 1 : 4, and 1 : 8 diluted samples (i.e., by dividing the VWF : Ag levels of neat sample by the dilution factors of 2, 4, and 8, respectively.

^e^Positive (+) and negative (−) reactions observed when neat and diluted samples were tested by using latex agglutination test.

VWF : Ag: von Willebrand factor antigen; LAT: latex agglutination test.

**Table 5:** Rheumatoid factor interference on high and low plasma pools.

Type of the plasma pool	Vol. of RF control serum (μL)^a^	Vol. of the plasma pool (μL)	RF conc. in plasma pool (IU/mL)	vWF LAT (titre)
High plasma pool	25	175	129	1 : 64
	30	180	147	1 : 128 (weak)
	40	200	172	1 : 128

Low plasma pool	10	990	10	1 : 2
	10	390	26	1 : 4
	10	190	52	1 : 8
	10	90	103	1 : 16
	10	70	129	1 : 32

^a^Rheumatoid factor control serum from MyBioSource, USA (1030 IU/mL), was used to spike plasma pools.

RF: rheumatoid factor; VWF LAT: von Willebrand factor latex agglutination test; vol., volume; conc., concentration.

**Table 6:** Crosstabulation of VWF antigen levels and qualitative VWF by latex agglutination test results.

	Qualitative VWF latex agglutination test (LAT score)				Total
	1+	2+	3+	4+	
VWF : Ag levels by immunoturbidimetric assay (%)					
<20	6	1	0	0	7
20–50	0	12	0	0	12
50–150	0	1	46	2	49
>150	0	0	15	49	64

Total	6	14	61	51	132

VWF: von Willebrand factor; VWF : Ag, von Willebrand factor antigen; LAT: latex agglutination test.

**Table 7:** Correlation of result interpretations by latex agglutination test and immunoturbidimetric assay.

Qualitative latex agglutination test (LAT score)	Semiquantitative latex agglutination test (titre)	VWF : Ag levels by immunoturbidimetric assay (%)
1+	1 : 2	<20
2+	1 : 4, 1 : 8	20–50
3+	1 : 16, 1 : 32	50–150
4+	≥64	>150

The proposed interpretation guide: normal level of VWF : Ag is indicated by LAT scores of 2+ and 3+ or titres from 1 : 8 to 1 : 32. Abnormal low VWF : Ag is indicated by LAT scores of 0, 1+, and 2+ or titres of 1 : 2 and 1 : 4. Abnormal high VWF : Ag is indicated by LAT score of 4+ or titres of ≥64.

LAT: latex agglutination test; VWF : Ag: von Willebrand factor antigen.
